# Contributions of changes in physical activity, sedentary time, diet and body weight to changes in cardiometabolic risk

**DOI:** 10.1186/s12966-021-01237-1

**Published:** 2021-12-20

**Authors:** Eivind Andersen, Hidde P. van der Ploeg, Willem van Mechelen, Cindy M. Gray, Nanette Mutrie, Femke van Nassau, Judith G. M. Jelsma, Annie S. Anderson, Marlene N. Silva, Hugo V. Pereira, Alex McConnachie, Naveed Sattar, Marit Sørensen, Øystein B. Røynesdal, Kate Hunt, Glyn C. Roberts, Sally Wyke, Jason M. R. Gill

**Affiliations:** 1 Institute for Sport and Social Science, Norwegian School of Sport Science, PO box 4014, Ullevål stadium, 0806 Oslo, Norway; 2 Amsterdam UMC, Vrije Universiteit Amsterdam, Department of Public and Occupational Health, Amsterdam Public Health research institute, Amsterdam, the Netherlands; 3 Institute of Health and Wellbeing, College of Social Sciences, University of Glasgow, Glasgow, UK; 4 Physical Activity for Health Research Centre, the University of Edinburgh, Edinburgh, UK; 5 Centre for Public Health Nutrition Research, University of Dundee, Dundee, UK; 6 CIDEFES, Faculdade de Educação Física e Desporto, Universidade Lusófona, Lisboa, Portugal; 7 CIPER, Faculdade de Motricidade Humana, Universidade de Lisboa, Lisboa, Portugal; 8 Robertson Centre for Biostatistics, Institute of Health and Wellbeing, University of Glasgow, Glasgow, UK; 9 Institute of Cardiovascular and Medical Sciences, University of Glasgow, Glasgow, UK; 10 Institute for Social Marketing and Health, University of Stirling, Stirling, UK

**Keywords:** Cardiovascular health, physical activity, sedentary time, body weight

## Abstract

**Background:**

Increased physical activity (PA), reduced time spent sedentary (SED), healthier diet and reduced body weight may all have a positive impact on cardiometabolic risk. The relative importance of change in each of these variables on cardiometabolic risk, however, is unclear. We therefore sought to investigate the relative contributions of changes in PA, SED, diet and body weight on cardiometabolic risk.

**Methods:**

This is a secondary analysis of data collected from the EuroFIT randomised controlled trial, which was a 12-week group-based lifestyle intervention for overweight middle-aged men delivered by coaches in football club stadia aiming to improve PA, SED, diet, and body weight. PA and SED were assessed by accelerometry, diet using the Dietary Instrument for Nutrition Education (DINE). An overall cardiometabolic risk score was derived from combining z-scores for glucose, HbA1c, insulin, lipids and blood pressure. In total, 707 men (from the overall cohort of 1113) with complete data for these variables at baseline and 12-month follow-up were included in the multivariable linear regression analyses.

**Results:**

In multivariable analyses, change in number of steps (explaining 5.1% of R^2^) and dietary factors (less alcohol, fatty and sugary food, and more fruit and vegetables) (together explaining 4.5% of R^2^), but not changes in standing time or SED, were significantly associated with change in body weight. Changes in number of steps (R^2^ = 1.7%), fatty food score (R^2^ = 2.4%), and sugary food score (R^2^ = 0.4%) were significantly associated with change in cardiometabolic risk score in univariable models. However, in multivariable models which included changes in weight as well as changes in steps and dietary variables, change in weight explained a substantially larger proportion of the change in cardiometabolic risk score, explaining 14.1% of R^2^ (out of an overall model R^2^ of 19.0%). When baseline (as well as change) values were also included in the model, 38.8% of R^2^ for change in cardiometabolic risk score was explained overall, with 14.1% of R^2^ still explained by change in weight.

**Conclusion:**

Change in body weight, together with baseline cardiometabolic risk explained most of the change in cardiometabolic risk. Thus, the benefits of increasing physical activity and improving diet on cardiometabolic risk appear to act largely via an effect on changes in body weight.

**Trial registration:**

International Standard Randomised Controlled Trials, ISRCTN-81935608. Registered 06052015. https://www.isrctn.com/ISRCTN81935608?q=&filters=recruitmentCountry:Portugal&sort=&offset=7&totalResults=92&page=1&pageSize=10&searchType=basic-search

**Supplementary Information:**

The online version contains supplementary material available at 10.1186/s12966-021-01237-1.

## Introduction

Increased physical activity (PA) and a healthy diet have consistently shown to be associated independently with lower risk of a range of chronic non-communicable diseases [1, 2], and have been a primary focus of the public health guidelines for many years [2–4]. PA (increased energy expenditure) and diet (caloric restriction) are also cornerstones in the prevention and management of overweight and obesity [5], which are strongly linked to risk of type 2 diabetes [6], cardiovascular diseases (CVD) [7] and overall mortality [8]. Data from randomised controlled trials (RCT) show that a combination of increased PA and dietary change is more effective for weight loss than each component alone [9], as well as being the most effective in reducing blood lipids and blood pressure [9]. The extent of change in CVD risk factors in response to a lifestyle intervention is related to the extent of body weight loss [10], and loss of body fat is likely to be an important mediator between changes in PA and diet and change in risk factors for cardiometabolic diseases [11]. However, both dietary improvements [12] and increases in PA [13] have been shown to change biomarkers of cardiometabolic risk, without a concomitant reduction in body weight.

More recently, there has been a large body of observational data suggesting that high levels of sedentary behaviour – defined as any waking behavior characterized by an energy expenditure ≤1.5 metabolic equivalents while in a seated, lying or reclining posture [14] – is associated with high risk of a range of adverse health outcomes, including CVD, type 2 diabetes and all-cause mortality [15, 16]. However, evidence regarding how much *changing* sedentary behaviour changes risk of these conditions is more limited. Short-term laboratory-based studies indicate that breaking up prolonged sitting with PA, can result in favourable metabolic changes [17–19], but effects of breaking up sitting time with standing are less clear [19–21]. Longer-term interventions aimed at reducing sedentary time by increasing standing time, have so far had a limited effect on biomarkers of cardiometabolic risk [22]. The extent to which reducing sitting time, as opposed to increasing PA, influences biomarkers of cardiometabolic risk is therefore presently uncertain and an important question for public health.

The European Fans in Training (EuroFIT) study was a large multi-country, men-only RCT that aimed to increase PA, reduce sedentary time, improve diet and reduce body weight over a follow-up period of 12 months [23, 24]. Over the course of the study, participants changed PA, sedentary time, diet and body weight by varying amounts, which provides the opportunity to understand more about the relative effects of each of these changes on biomarkers of cardiometabolic risk. The aim of the research reported in this paper was therefore, firstly, to investigate the associations between changes in PA, sedentary time and dietary intake on changes in body weight and, secondly, to investigate the associations between changes in PA, sedentary time and dietary intake, and the associated changes in body weight, on changes in a cardiometabolic risk score, in men who participated in the EuroFIT RCT. We additionally sought to investigate the extent to which any associations of changes in PA, sedentary time and dietary intake and changes in cardiometabolic risk score were mediated by changes in bodyweight.

## Methods

### Study design

This study is a secondary analysis based on data collected from the EuroFIT lifestyle intervention study (ISRCTN-81935608), a pragmatic two-arm RCT conducted in 2016 and 2017 in 15 professional football clubs in England (five clubs), the Netherlands (four clubs), Norway (three clubs), and Portugal (three clubs). Ethics committees in each of the four countries approved the study [24], and written informed consent was obtained from all participants. The study design, intervention delivery protocol and methods of EuroFIT have been described in detail previously [24, 25]. Briefly, the EuroFIT intervention aimed to support men to become gradually more physically active, reduce their sedentary time, improve their diet, and to maintain these changes to at least 12 months after baseline. We trained coaches at the football clubs to deliver the intervention to male fans of the clubs in an accessible style, including encouraging positive banter, making sessions enjoyable, promoting a ‘team’ environment, and using interactional approaches congruent with other male contexts. The program was delivered at club stadia, to groups of 15-20 men over 12 weekly, 90-minute sessions that combined the interactive development of self-regulation skills via a toolkit of behavior change techniques (including goal setting and review, action planning, self-monitoring, and provision of information about health and emotional benefits of change), with graded group-based moderate intensity PA. In addition, men were provided with a novel validated, pocket-worn device (SitFIT) to enable self-monitoring of sedentary time and physical activity [26]. Peer support was also encouraged via social media platforms, and an interactive social team-based step-challenge app (MatchFIT) [23]. An additional reunion meeting was scheduled 6-9 months after the start of the program. Men allocated to the comparison group were offered the opportunity to take part in the EuroFIT intervention after the 12-month measures. The analyses for this paper use data from the intervention group and the comparison group merged into a single cohort dataset.

### Recruitment and participants

The participant recruitment was led by the football clubs and involved multiple strategies, including e-mail invitations to club members, website articles, social network posting with club celebrity endorsements, match-day recruitment and features in local press. Online eligibility screening collected contact details: age, self-reported height and body weight, preferred football club, and current participation in health promotion programs at the club from the men. A follow-up telephone call administered the adapted Physical Activity Readiness Questionnaire-Plus questionnaire (PAR-Q+) [27] and asked if men were willing to consent to randomisation and to wearing an activity monitor for one week, on three occasions (baseline, post-program and 12 month follow-up). Men were eligible if they were aged 30-65, had a body mass index (BMI) of ≥27 kg∙m^–2^ based on self-reported height and body weight and consented to study procedures. Men were excluded if they reported a contraindication to moderate- to vigorous PA in the PARQ+, participated in an existing health promotion program at the club, or were unable to provide at least four days of usable activity monitor data at baseline. In this secondary analysis, only men with valid accelerometer recordings and blood samples at baseline and 12-month follow-up were included.

### Measurements

#### Objective PA and sedentary time

Free-living PA and sedentary time were assessed using the activPAL monitor (model activPAL^TM^ micro; PAL Technologies Ltd, Glasgow, UK), a thigh-worn tri-axial inclinometer which provides an objective measure of sitting, standing and PA using proprietary software, and has been found to have good measurement properties to assess sedentary, standing, and stepping time and postural transitions in adults [28–30]. Participants were asked to wear the activPAL monitor for 24-hours per day on seven consecutive days at baseline, post-program (12 weeks), and 12 months as previously described [23].

#### Self-reported dietary data

Diet was self-reported using an adapted Dietary Instrument for Nutrition Education (DINE) [31]. The current approach queried intake of the main sources of dietary fat and sugar (cheese, burgers or sausages, beef, pork or lamb, fried food, chips or French fries, bacon or ham or pate, savoury pies, pasties, sausage rolls and pork pies, savoury snacks, consumption of fruit, vegetables (not potatoes), chocolate, sweets, biscuits, sugary drinks (fizzy drinks, diluting/ fruit juice) and milk) and scores were calculated based on frequency of consumption. Fatty food scores could range from 6.5 to 66.5, with each unit broadly equivalent to an additional portion of crisps per week; sugary food scores could range from 3 to 18, with each unit broadly equivalent to an additional biscuit or chocolate bar per day; and fruit and vegetable scores could range from 1 to 12, with each unit broadly equivalent to an additional portion of fruit or vegetables per day. Alcohol intake was assessed using a 7-day recall questionnaire.

#### Self-reported smoking data

Participants were asked about their current smoking status and whether they had ever smoked.

#### Objective physical measures

Body weight was measured, with men wearing light clothing, using an electronic flat scale (Tanita HD366). Body height was measured at baseline only, without shoes, using a stadiometer (Leicester Height Measure). BMI was calculated as body weight (kilograms) divided by the square of body height in meters (kg∙m^–2^). Waist circumference was measured twice (three times, if the first two measurements differed by >0.5 cm) using a tape measure (Seca 201) and the mean calculated over the nearest two measurements. Blood pressure (mmHg) was measured with a blood pressure monitor (Omron 705-CPII) after 5 minutes sitting still. If the blood pressure was above normal range, two extra measures was taken.

#### Blood collection

Blood samples were collected after a minimum six hours fasting. Samples were stored at 4°C and processed within 24h, and then frozen at -80°C. Biochemistry tests for fasting serum glucose, total serum cholesterol, high-density lipoprotein cholesterol (HDL-C), triglycerides, hemoglobin A1c (HbA1c) (c311, Roche Diagnostics, Burgess Hill, UK) and insulin immunoassays (e411, Roche Diagnostics, Burgess Hill, UK) were run on clinically validated automated platforms. All tests used manufacturers’ reagents, calibrators and quality control materials. All coefficients of variation for quality controls were <5%.

### Statistical analysis

Statistical analysis was performed using Minitab (version 19, State College, PA). ActivPAL data were processed using proprietary software developed by PAL Technologies, and summarized as daily time spent sedentary, standing and stepping and number of steps taken per day [23]. Dietary variables were summarized into a fatty food score (range 6.5-66.5), sugary food score (range 3-18), fruit and vegetable intake score [1–12] and, alcohol intake (units per week) [23].

Cardiometabolic risk factor variables related to glycaemia (HbA1c, fasting plasma glucose), lipids/insulin resistance (HDL-C, triglycerides, total cholesterol and insulin) and blood pressure (systolic and diastolic blood pressure) were summarised into a single cardiometabolic risk score as previously described [32]. These variables were chosen as established biomarkers with strong associations with incident cardiovascular and metabolic disease [33], and the cardiometabolic risk score was calculated as (the z-score for the sum of z-scores for fasting glucose, fasting insulin, HbA1c, total cholesterol, (-)HDL-C, triglyceride, systolic blood pressure and diastolic blood pressure)*10. A negative value for the HDL-C z-score was used due to the inverse association between HDL-C concentration and cardiovascular disease risk. Thus, the mean cardiometabolic risk score for the group is 0, a difference of 10 represents 1 standard deviation (SD) difference from the mean, and a change of 10 in the cardiometabolic risk score represents a 1 SD change in response to the intervention from baseline. A higher cardiometabolic risk score value denotes higher risk.

Baseline characteristics and change between baseline and 12 months for the intervention group, comparison group, and both groups combined, are reported as mean, SD and range. Differences between the intervention group and comparison group for changes between baseline and 12 months were assessed by unpaired t-tests. A number of univariable associations were calculated by linear regression analyses of changes in PA variables (standing time, stepping time, number of steps), sedentary time, dietary variables (fatty food score, sugary food score, fruit and vegetable score, alcohol intake), respectively, and changes in body weight and cardiometabolic risk score (Model 1 in Tables 3 and 4, respectively). Thereafter, multivariable linear regression analyses were performed to establish the relative contributions of changes in PA and dietary variables, to changes in body weight and cardiometabolic risk score (Model 2 in Tables 5 and 6, respectively). These models could not include both change in step count and change in minutes of stepping due to collinearity; change in step count was included due to its stronger univariable associations with change in body weight and change in cardiometabolic risk score. To determine the relative contributions of changes in PA, of changes in dietary variables and of change in body weight to change in cardiometabolic risk score, change in body weight was then added to Model 2 with change in cardiometabolic risk score as the outcome (Model 3). To determine the extent to which baseline values, change in smoking status, and any intervention effect not captured in the measured outcome variables might have influenced the relationship with changes in body weight and cardiometabolic risk score, a final model adding baseline values for all included predictor variables and control or intervention group status was then run for Model 2 with body weight as the outcome and for Model 3 with cardiometabolic risk score as the outcome. Finally, a mediation analysis was performed to estimate the extent to which any associations of changes in PA, sedentary time and dietary intake and changes in cardiometabolic risk score were mediated by changes in bodyweight, with statistical significance of the mediation (indirect) effects calculated using the Sobel test [34]. P < 0.05 was accepted as an indicator of statistical significance.

## Results

Participants were recruited between September 19, 2015, and February 2, 2016. Main reasons for exclusion for men who showed interest in the trial were BMI *<*27 kg∙m^–2^ (42.4%) and because the study had reached the maximum number of participants at their club of interest (39.3%). A total of 1113 men constituted the sampling frame for this study. Participants spanned all sociodemographic groups, had at least 12 years of education, and the majority were in full-time work, and were married or living with a partner [23]. A final sample of 707 men was included in this secondary analysis, after excluding those with missing ActivPAL data (n=198), or missing cardiometabolic risk score data (from voluntary blood samples) (n=322), which included 114 men who were missing both. Excluded men were compared to included men, and were of the same age (45.4±8.5 years vs. 46.0±9.0 years, p=0.27), but somewhat heavier (107.4±18.5 kg vs. 105.1±17.0 kg, p=0.039, and with a BMI 33.7±5.2 kg∙m^–2^ vs. 33.0±4.3 kg∙m^–2^, p=0.014). In total, 699 participants reported their smoking status at baseline: 339 (170 Intervention, 177 Comparison) were never smokers, 253 (125 Intervention, 128 Comparison) were ex-smokers and 107 (50 Intervention, 57 Comparison) were current smokers.

At baseline, participants mean daily step count was 8604±3251 steps per day (mean ± SD), sedentary time was 618±113 min^.^day^-1^, body weight 105.1±17.0 kg, waist circumference 110.7±11.8 cm and BMI 33.0±4.3 kg∙m^–2^ (Table 1). Mean values for diet, alcohol and cardiometabolic biomarkers were all in the normal range. However, a large degree of inter-individual variation was observed for all variables (Table 1). Except for sedentary time and standing time, the intervention group had improved significantly more at the 12 month follow-up on all measured variables compared to the comparison group (Table 2). As for the baseline results, a large inter-individual variation was observed for the change from baseline to 12 months for all variables (Table 2).

**Table 1:** Baseline characteristics of the study population

	Intervention (n = 349)			Comparison (n = 358)			Overall (n = 707)		
	Mean	SD	Range	Mean	SD	Range	Mean	SD	Range
Age (years)	46.0	9.0	30.4 – 65.0	45.9	9.1	30.2 – 65.1	46.0	9.0	30.2 – 65.1
Body weight (kg)	104.1	16.1	71.1 – 182.9	106.1	17.3	72.5 – 185.8	105.1	17.0	71.1 – 185.8
BMI (kg^.^m^-2^)	32.8	4.2	25.1 – 51.8	33.2	4.4	24.9 – 56.7	33.0	4.3	24.9 – 56.7
Waist circumference (cm)	110.3	11.3	84.6 – 157.3	111.1	12.2	85.1 – 158.2	110.7	11.8	84.6 – 158.2
Number of steps (steps per day)	8627	3334	2014 – 21886	8581	3272	1875 – 24343	8604	3251	1875 – 24343
Stepping time (min^.^day^-1^)	108	37	27 – 240	109	39	27 – 323	109	38	27 – 324
Standing time (min^.^day^-1^)	254	86	70 – 523	248	84	80 – 596	251	85	70 – 596
Sedentary time (min^.^day^-1^)	613	111	323 – 937	622	115	307 – 1057	618	113	307 – 1057
Fatty food score (range 6.5-66.5)	18.6	5.6	0 – 42	19.3	6.0	0 – 45	18.9	5.8	0 – 45
Sugary food score (range 3-18)	5.6	3.1	0 – 18	5.7	3.4	0 – 18	5.7	3.2	0 – 18
Fruit and vegetable score (range 1-12)	3.9	2.9	0 – 12	3.8	2.6	0 – 12	3.9	2.7	0 – 12
Alcohol intake (units^.^week^-1^)	6.5	8.4	0 – 56	5.9	7.3	0 – 42	6.2	7.9	0 – 56
Fasting glucose (mmol^.^l^-1^)	4.5	0.8	2.0 – 9.6	4.7	1.6	2.2 – 19.0	4.6	1.3	2.0 – 19.0
Fasting insulin (mU^.^L^-1^)	18.0	20.1	0.9 – 155.8	19.5	24.3	1.5 – 328.1	18.7	22.3	0.9 – 328.1
HbA1c (mmol^.^mol^-1^)	34.7	7.0	19.8 – 73.8	35.6	9.4	23.8 – 109.1	35.2	8.3	19.8 – 109.1
Total cholesterol (mmol^.^l^-1^)	4.9	1.1	1.9 – 11.7	4.9	1.1	2.4 – 9.4	4.9	1.1	1.9 – 11.7
HDL cholesterol (mmol^.^l^-1^)	1.1	0.3	0.4 – 2.7	1.0	0.3	0.4 – 2.2	1.0	0.3	0.4 – 2.7
Triglycerides (mmol^.^l^-1^)	2.1	1.6	0.5 – 14.3	2.3	1.9	0.6 – 25.0	2.2	1.7	0.5 – 25.0
Systolic blood pressure (mmHg)	133.5	13.6	105 – 187	134.6	14.6	102 – 203	134.0	14.1	102 – 203
Diastolic blood pressure (mmHg)	84.7	9.9	53 – 113	85.4	9.4	59 – 121	85.1	9.6	53 – 121
Cardiometabolic risk score^a^	-0.87	8.61	-25.32 – 43.60	0.85	11.14	-20.44 – 78.06	0.00	10.00	-25.32 – 78.06

^a^Cardiometabolic risk score calculated as (the z-score for the sum of z-scores for fasting glucose, fasting insulin, HbA1c, total cholesterol, HDL cholesterol^#^, triglyceride, blood pressure and diastolic blood pressure)*10. ^#^negative value for HDL cholesterol z-score used. Thus 0 is the mean risk score for the group, and a difference of 10 represents 1 SD difference from the mean. A higher value denotes higher risk.

**Table 2:** Change between baseline and 12-months in response to the EuroFIT intervention

	Intervention (n = 349)			Comparison (n = 358)			Overall (n = 707)		
	Mean	SD	Range	Mean	SD	Range	Mean	SD	Range
Body weight (kg)	-3.0*	6.5	-39.2 – 15.5	-0.6	5.0	-22.2 – 12.9	-1.8	5.9	-39.2 – 15.5
Waist circumference (cm)	-3.3*	6.7	-28.6 – 34.2	-0.5	5.6	-27.1 – 27.4	-1.9	6.3	-28.6 – 34.2
Number of steps (steps per day)	754*	3123	-7929 - 22656	-10	2342	-12315 – 13463	367	2780	-12315 - 22656
Stepping time (min^.^day^-1^)	7.0*	34.3	-117 – 232	-0.3	26.1	-125 – 117	3.3	30.6	-125 – 232
Standing time (min^.^day^-1^)	-6.5	78.1	-304 – 327	-1.6	64.8	-199.5 – 197.8	-4.0	71.7	-304.0 – 326.8
Sedentary time (min^.^day^-1^)	-8.6	100.9	-387.9 – 455.1	-5.8	90.9	-351.5 – 285.0	-7.2	96.0	-387.9 – 455.1
Fatty food score	-1.9*	5.7	-26.5 – 17.5	-0.8	5.8	-22.0 – 17.0	-1.3	5.8	-26.5 – 17.5
Sugary food score	-1.1*	2.9	-13 – 12	-0.5	3.1	-13 – 14	-0.8	3.0	-13 – 14
Fruit and vegetable score	1.1*	3.4	-9 – 10	0.2	3.0	-10 – 12	0.7	3.3	-10 – 12
Alcohol intake (units^.^week^-1^)	-1.5	5.9	-36 – 39	-0.2	5.1	-30 – 28	-0.8	5.6	-36 – 39
Cardiometabolic risk score ^a^	-2.05*	7.75	-47.59 – 21.44	-0.84	8.35	-57.60 – 19.52	-1.43	8.07	-57.60 – 21.44

*p < 0.05 compared to change in Comparison group.

^a^difference of 10 in cardiometabolic risk score represents a 1 SD change in response to the intervention from baseline.

In univariable analyses, changes from baseline to 12 months in the number of steps, stepping time, sedentary time and fatty food, sugary food and alcohol intake were significantly associated with change in body weight (Table 3). However, change in standing time and fruit and vegetable score was not associated with change in body weight. Change in PA was more strongly associated with change in body weight (R^2^ of 5.1% for change in steps per day and 3.6% for change in stepping time) than change in sedentary time was with body weight (R^2^ of 0.8%), with an increase in 1000 steps per day being associated with a decrease of 0.48 kg in body weight. Changes in dietary variables explained broadly comparable proportions of the variance in change in body weight as change in steps. Changes between baseline and 12 months in number of steps, stepping time, fatty food score, sugary food score, alcohol intake, and body weight and waist circumference were all significantly associated with change in cardiometabolic risk score in univariable analyses, while sedentary time, standing time and intake of fruit and vegetables were not significantly related to change in cardiometabolic risk score (Table 4). However, changes in body weight (R^2^ of 18.0%) and waist circumference (R^2^ of 12.0%) explained a relatively much larger proportion of the change in the cardiometabolic risk score than the PA and dietary variables (≤3% variance explained) (Table 4). There was no significant association between change in smoking status and either change in weight (R^2^ = 0.1%, p = 0.34) or change in cardiometabolic risk score (R^2^ = 0.2%, p = 0.29). This may be due to the small proportion of participants who changed smoking status over the intervention period (<4%); 20 participants (9 Intervention, 11 Comparison) stopped smoking and six ex-smokers (4 Intervention, 2 Comparison) restarted smoking during the intervention period. Change in smoking status was therefore not included in the multivariable analysis models.

**Table 3:** Univariable associations between changes between baseline and 12-months in PA, sedentary time and diet, and change between baseline and 12-month in body weight in the EuroFIT study

Model 1 (univariable) (n = 707)				
		Difference in change in weight from baseline, associated with stated increase in predictor		
Predictor (change from baseline)	Increase in predictor	Estimate	95% CI	R^2^
Number of steps (steps per day)^a^	1000 steps per day	-0.48	(-0.63, -0.32)	5.1%
Stepping time^a^	10 minutes per day	-0.36	(-0.50, -0.22)	3.6%
Standing time	10 minutes per day	-0.01	(-0.07, 0.05)	0.0%
Sedentary time^b^	10 minutes per day	0.05	(0.008, 0.099)	0.8%
Fatty food score^a^	1 point	0.20	(0.13, 0.27)	4.0%
Sugary food score^a^	1 point	0.26	(0.11, 0.40)	1.8%
Fruit and vegetable score^a^	1 point	-0.11	(-0.25, 0.02)	0.4%
Alcohol intake^b^	1 unit per week	0.08	(0.01, 0.16)	0.7%

^a^P<0.01, ^b^P<0.05

**Table 4:** Univariable associations between changes between baseline and 12-month in PA, sedentary time, diet, bodyweight and waist circumference, and change between baseline and 12-month in cardiometabolic risk score in the EuroFIT study

Model 1 (univariable) (n = 707)				
		Difference in change in cardiometabolic risk score from baseline, associated with stated increase in predictor		
Predictor (change from baseline)	Increase in predictor	Estimate	95% CI	R^2^
Number of steps^a^	1000 steps per day	-0.38	(-0.59, -0.17)	1.7%
Stepping time^a^	10 minutes per day	-0.30	(-0.49, -0.01)	1.3%
Standing time	10 minutes per day	-0.026	(-0.11, 0.057)	0.0%
Sedentary time	10 minutes per day	0.033	(-0.029, 0.095)	0.2%
Fatty food score^a^	1 point	0.23	(0.13, 0.34)	2.8%
Sugary food score^a^	1 point	0.29	(0.09, 0.49)	1.2%
Fruit and vegetable score	1 point	-0.08	(-0.26, 0.10)	0.1%
Alcohol intake^b^	1 unit per week	0.09	(0.02, 0.19)	0.4%
Body weight^a^	1 kg	0.58	(0.49, 0.67)	18.0%
Waist circumference^a^	1 cm	0.44	(0.35, 0.53)	12.0%

^a^P<0.01, ^b^P<0.05

In multivariable analysis including PA, sedentary time and dietary variables in the same model (Table 5, Model 2), changes in number of steps, fatty food score, sugary food score and fruit and vegetables score, but not sedentary time, standing time and alcohol intake, were significantly associated with change in body weight. This model explained 10.3% of the variance in change in body weight. When baseline values and group membership were added to the model (Table 5, Model 3), the aforementioned variables remained significantly associated with body weight change. Baseline fatty and sugary foods score, baseline body weight and group membership (larger change in intervention group) were also significantly associated with change in body weight. The addition of baseline values and group membership to the model, explained an additional 6.9% of the variance in body weight change.

**Table 5:** Multivariable associations between changes between baseline and 12-month in PA, sedentary time, and diet, and change between baseline and 12-month in body weight in the EuroFIT study

Model 2 (multivariable – changes in PA variables and diet) (n = 707)					
	β-coefficient	95% CI	p	R^2^	Model R^2^
Change in number of steps (steps per day)^a^	-0.42	(-0.58, -0.26)	**<0.0005**	5.1%	10.3
Change in standing time (min^.^day^-1^)^a^	0.05	(-0.02, 0.13)	0.145	0.2%	
Change in sedentary time (min^.^day^-1^)^a^	0.03	(-0.02, 0.10)	0.216	0.2%	
Change in fatty food score	0.14	(0.07, 0.22)	**<0.0005**	3.1%	
Change in sugary food score	0.20	(0.05, 0.35)	**0.006**	0.6%	
Change in fruit and vegetable score	-0.17	(-0.30, -0.03)	**0.011**	0.8%	
Change in alcohol intake (units^.^week^-1^)	0.05	(-0.02, 0.12)	0.184	0.3%	
**Model 3 (multivariable, Model 2 plus baseline values and group membership)** **(n = 707)**					
Change in number of steps (steps per day)^a^	-0.44	(-0.62, -0.27)	**<0.0005**	5.1%	17.2
Change in standing time (min^.^day^-1^)^a^	0.04	(-0.04, 0.12)	0.343	0.2%	
Change in sedentary time (min^.^day^-1^)^a^	0.02	(-0.04, 0.09)	0.464	0.2%	
Change in fatty food score	0.20	(0.11, 0.28)	**<0.0005**	3.1%	
Change in sugary food score	0.30	(0.11, 0.49)	**0.001**	0.6%	
Change in fruit and vegetable score	-0.18	(-0.33, -0.02)	**0.021**	0.8%	
Change in alcohol intake (units^.^week^-1^)	0.05	(-0.02, 0.14)	0.183	0.2%	
Baseline number of steps (steps per day)^a^	-0.14	(-0.30, 0.007)	0.061	0.2%	
Baseline standing time (min^.^day^-1^)^a^	-0.01	(-0.09, 0.07)	0.765	0.1%	
Baseline sedentary time (min^.^day^-1^)^a^	-0.01	(-0.08, 0.05)	0.602	0%	
Baseline fatty food score	0.14	(0.05, 0.23)	**0.002**	1.8%	
Baseline sugary food score	0.17	(0.005, 0.353)	**0.043**	0.7%	
Baseline fruit and vegetable score	-0.06	(-0.24, 0.11)	0.490	0.1%	
Baseline alcohol intake (units^.^week^-1^)	0.01	(-0.04, 0.07)	0.610	0%	
Baseline body weight (kg)	-0.06	(-0.08,-0.03)	**<0.0005**	2.7%	
Group (1 intervention, 2 comparison)	1.44	(0.58, 2.29)	**0.001**	1.3%	

^a^Data presented as β-coefficient and 95% CI for change in weight per 1000 steps per day, and 10 min change in standing time and sedentary time.

Table 6 shows that, in multivariable analyses, changes in number of steps and fatty and sugary food scores, but not changes in sedentary time, standing time, fruit and vegetable score or alcohol intake, were significantly associated with change in cardiometabolic risk score, explaining 3.8% of the change in cardiometabolic risk score (Model 2). However, when change in body weight was added to the model, its contribution to change in cardiometabolic risk score was an order of magnitude higher than that of changes in PA and dietary variables (14.1% of the 19.0% R^2^ value in model 3), the association between changes in number of steps and fatty and sugary food score and changes in cardiometabolic risk score observed in model 2 was lost. When baseline values and group membership were added to the model (Model 4), changes in body weight and baseline cardiometabolic risk score were by far the most important drivers of change in the cardiometabolic risk score, explaining 32.1% of the 38.8% R^2^ value in model 4, with change in sugary food score also a making small, but statistically significant contribution.

**Table 6:** Multivariable associations between changes between baseline and 12-month in PA, sedentary time, diet and body weight, and change between baseline and 12-month in cardiometabolic risk score in the EuroFIT study

Model 2 (multivariable – changes in PA variables and diet) (n = 707)					
	β-coefficient	95% CI	p	R^2^	Model R^2^
Change in number of steps (steps per day)^a^	-0.33	(-0.55, -0.10)	**0.002**	1.7%	4.9%
Change in standing time (min^.^day^-1^)^a^	0.00	(-0.01, 0.01)	0.940	0.0%	
Change in sedentary time (min^.^day^-1^)^a^	0.000	(-0.009, 0.008)	0.929	0.0%	
Change in fatty food score	0.19	(0.08, 0.29)	**0.001**	2.4%	
Change in sugary food score	0.22	(0.01, 0.43)	**0.042**	0.4%	
Change in fruit and vegetable score	-0.14	(-0.32, 0.05)	0.144	0.3%	
Change in alcohol intake (units^.^week^-1^)	0.04	(-0.06, 0.14)	0.425	0.1%	
**Model 3 (multivariable – Model 2 plus change in body weight)** **(n = 707)**					
Change in number of steps (steps per day)^a^	-0.10	(-0.31, 0.12)	0.368	1.7%	19.0%
Change in standing time (min^.^day^-1^)^a^	0.00	(-0.01, 0.01)	0.491	0.0%	
Change in sedentary time (min^.^day^-1^)^a^	0.00	(-0.01, 0.01)	0.541	0.0%	
Change in fatty food score	0.10	(0.01, 0.20)	**0.038**	2.4%	
Change in sugary food score	0.11	(-0.09, 0.30)	0.289	0.4%	
Change in fruit and vegetable score	-0.05	(-0.22, 0.13)	0.600	0.3%	
Change in alcohol intake (units^.^week^-1^)	0.02	(-0.08, 0.11)	0.757	0.1%	
Change in body weight (kg)	0.54	(0.44, 0.64)	**<0.0005**	14.1%	
**Model 4 (multivariable, Model 3 plus baseline values and group membership)** **(n = 707)**					
Change in number of steps (steps per day)^a^	-0.13	(-0.34, -0.07)	0.19	1.78%	37.2%
Change in standing time (min^.^day^-1^)^a^	-0.01	(-0.06, 0.03)	0.629	0.1%	
Change in sedentary time (min^.^day^-1^)^a^	-0.02	(-0.06, 0.01)	0.200	0.0%	
Change in fatty food score	0.01	(-0.03, 0.06)	0.554	1.7%	
Change in sugary food score	0.12	(0.01, 0.22)	**0.026**	0.6%	
Change in fruit and vegetable score	-0.02	(-0.11, 0.06)	0.589	0.3%	
Change in alcohol intake (units.week^-1^)	0.02	(-0.02, 0.07)	0.261	0.3%	
Change in body weight (kg)	0.23	(0.19, 0.28)	**<0.0005**	15.3%	
Baseline number of steps (steps per day)^a^	-0.07	(-0.16, 0.13)	0.094	0.2%	
Baseline standing time (min^.^day^-1^)^a^	0.015	(-0.03, 0.06)	0.532	0.0%	
Baseline sedentary time (min^.^day^-1^)^a^	0.001	(-0.03, 0.04)	0.962	0.0%	
Baseline fatty food score	0.03	(-0.02, 0.08)	**0.231**	0.4%	
Baseline sugary food score	0.08	(-0.01, 0.18)	0.082	0.4%	
Baseline fruit and vegetable score	0.06	(-0.04, 0.16)	0.236	0.1%	
Baseline alcohol intake (units^.^week^-1^)	-0.01	(-0.04, 0.02)	0.561	0.0%	
Baseline body weight (kg)	0.011	(-0.003, 0.025)	0.135	0.2%	
Baseline cardiometabolic risk score	-0.36	(-0.41, -0.30)	**<0.0005**	17.5%	
Group (1 intervention, 2 comparison)	0.06	(-0.43, 0.55)	0.812	0.0%	

^a^Data presented as β-coefficient and 95% CI for change in cardiometabolic risk score per 1000 steps per day, and 10 min change in standing time and sedentary time.

**Fig. 1:**
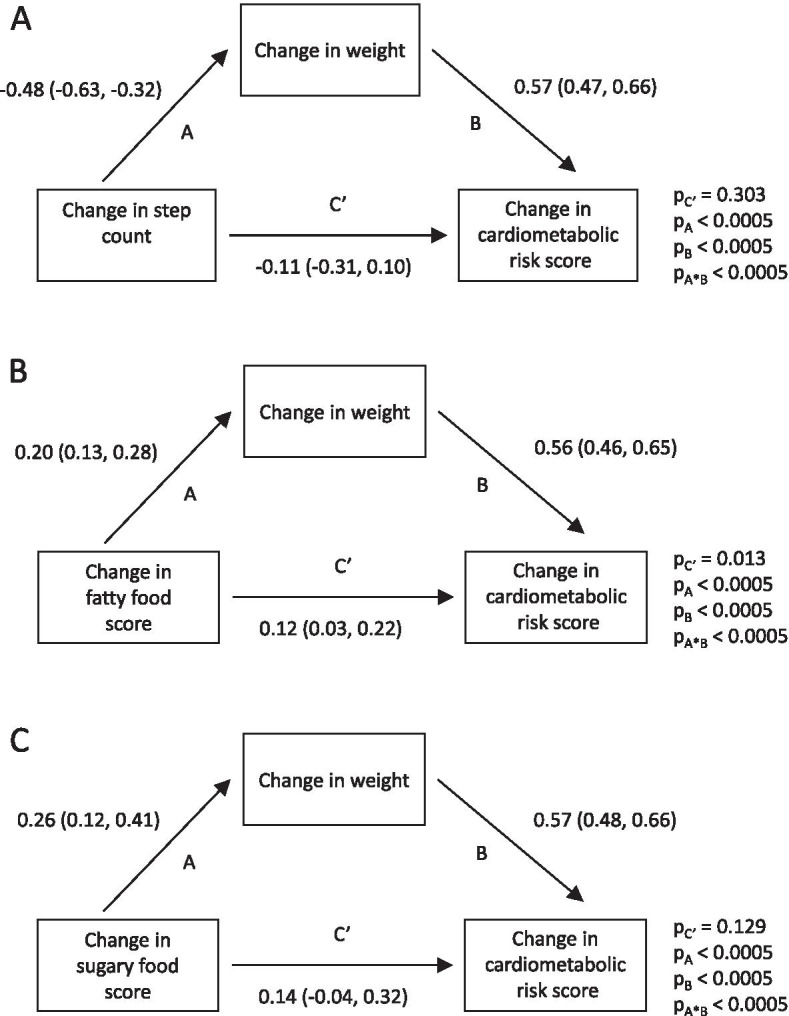
Path diagrams indicating the indirect effect of change in weight on the association between A) change in step count, B) change in fatty food score, and C) change in sugary food score, and change in cardiometabolic risk score in the EuroFIT study. For all three behavoiural variables a significant assocation between their change and change in cardiometabolic risk score was observed (see Table 4)

For behavioural variables where a change was associated with a change in cardiometabolic risk score in univariable analyses (change in steps, fatty food score, and sugary food score; see Table 4), mediation analyses were performed to determine the extent to which these associations were mediated by changes in body weight (Fig. 1). For change in step count, ~71% of the association with change in cardiometabolic risk score (overall -0.38 SD change in cardiometabolic risk score per 1000 step per day increase, with the indirect effect of change in weight accounting for -0.27 SD of this change) was mediated by the indirect effect of change in weight (p < 0.0005). For changes in fatty and sugary food scores, ~48% (0.11 SD out of 0.23 SD change in cardiometabolic risk score per unit change) and ~52% (0.15 SD out of 0.29 SD change in cardiometabolic risk score per unit change), respectively, of the associations with cardiometabolic risk score were mediated by change in weight (p < 0.0005, for both). The direct effect, independent of change in weight, was only statistically significant for change in fatty food score (p = 0.013).

## Discussion

In this study, we aimed to determine the independent contributions of changes in PA, sedentary behaviour, and diet on change in body weight (and waist circumference), and changes in PA, sedentary behaviour, and body weight on change in cardiometabolic risk, in men who participated in the EuroFIT study. Two major findings emerged from this study. Firstly, both in univariable and in multivariable analyses, changes in number of steps and dietary variables (fatty and sugary food scores, fruit and vegetable score and alcohol intake), but not changes in sedentary time or standing time, were significantly associated with change in body weight. Secondly, changes in number of steps, body weight and sugar intake, and high baseline cardiometabolic risk were all significantly associated with change in the cardiometabolic risk score in the full multivariable model. However, changes in sedentary time, standing time and other dietary variables were not significantly associated in the full multivariable model with change in the cardiometabolic risk score. Perhaps more importantly, of the variables included in the models, change in body weight and baseline cardiometabolic risk score were, by far, the most important predictors of the change in cardiometabolic risk score. Mediation analysis also revealed that the associations of changes in PA and dietary intake with changes in cardiometabolic risk score were substantially mediated by changes in weight. Our results therefore imply that the benefits of increasing PA and improving diet on cardiometabolic risk may act largely via effects on body weight.

Our finding that change in sedentary time was not associated with change in body weight when controlling for change in PA and diet is in contrast to findings of most published cross-sectional studies [35–38]. However, in cross-sectional studies, the direction of causality cannot be ascertained and there is plausible data to suggest that higher BMI can result in higher sedentary time, rather than *vice versa* [39, 40]. Three systematic reviews of prospective studies all concluded that the evidence for an association of sedentary time with weight gain, or the risk of obesity, was equivocal [41–43]. Furthermore, a recent meta-analysis on data from 23 prospective cohort studies concluded that there were small, inconsistent and non-significant associations between sedentary time and body weight [44], which are also in line with our findings. However, no RCT has been conducted and the effect of reducing sedentary time on body weight is unclear. It might be that any possible harmful effects of sedentary behaviour on health are mediated through mechanisms other than change in body weight.

Both increased PA (0.03 SD change in cardiometabolic risk score per 1000 step increase) and reduced fat and sugar intake (0.02 SD change per unit change in fatty food or sugary food score) were significantly associated with a reduced cardiometabolic risk. This finding is in line with previous results [9]. However, the lack of an association between change in sedentary time and change in cardiometabolic risk is in contrast to most published studies [15, 43, 45, 46]. Most of the prospective sedentary research is, however, based on self-reported data, which are prone to recall bias and subject to social desirability, especially sitting time [47]. In addition, many studies have used time spent watching TV as an indicator of total sedentary time, which may be confounded by (unhealthy) eating habits, socio-economic status and mental health [48–50]. The studies that have used objective methods (accelerometry) to measure sedentary behaviour have used waist-worn accelerometers, which do not distinguish between standing and sitting, but rather report a general lack of ambulatory movement (no acceleration) [51]. In a validation study by Kozey-Keadle et al. (2010), the correlation between a waist worn Actigraph GT3X sedentary time and direct observation of sitting time was only R^2^=0.39 compared to R^2^=0.94 for the thigh worn activPAL [52]. Hence, a thigh-worn accelerometer, used in the present study, is better able to elucidate the association between changes in sedentary time and change in cardiometabolic risk. Another crucial point is that these epidemiological studies looked at sedentary behaviour at a single time point and how this was associated with risk of adverse health outcomes in the future, and not whether increasing or decreasing sedentary time over time between changed risk. The EuroFIT study design (RCT) allowed us to investigate whether change in sedentary time was related to change in cardiometabolic risk over a 12-month period. Few experimental studies looking at the cardiometabolic health benefits of changing sedentary behaviour have been conducted and the findings of these experimental studies are inconsistent and with small sample sizes and/or short duration (three months or less) [53–58]. An exception is the methodologically rigorous “Stand Up Victoria” trial, which were able to produce large reductions in sitting time, but with only modest improvements to cardiometabolic health [22].

The associations between changes in PA and sugar intake and change in cardiometabolic risk remained after adjustment for baseline values and change in body weight, although their contribution was rather small. By far, the most important factors explaining change in cardiometabolic risk were baseline cardiometabolic risk and change in body weight in the fully adjusted model. A high baseline cardiometabolic risk score provides more room for improvement in response to a lifestyle intervention. Perhaps more unexpected was the relatively strong contribution of change in body weight to change in cardiovascular risk score, compared to the PA and dietary variables. To our knowledge, there are no other published studies that have investigated the relative contribution of change in body weight (or other measures of body weight) to a change in cardiometabolic risk score, adjusted for changes in PA and diet. Rather, other studies have looked at the associations between changes in PA, diet and cardiometabolic risk and treated change in adiposity (e.g. BMI and waist circumference) solely as a confounding factor. Most [11, 37, 38, 59–67], but not all studies [11, 59, 60], find associations between changes in PA and sedentary time and change in cardiometabolic risk, after adjusting for change in body weight. A novel finding of our study is thus the seemingly greater importance of a reduction in body weight on cardiometabolic risk, compared to change in PA, sedentary time and diet. A possible mechanistic route is that a reduction in body fat, most likely caused by changes in PA and diet, were related to changes in cardiometabolic risk factors through a reduced secretion of free fatty acids and an accompanied reduced inflammation and improved insulin sensitivity [68].

The main strength of this study is that it provides novel experimental and prospective evidence of the association between *changes* in PA, sedentary time, diet and body weight and *change* in cardiometabolic risk. Furthermore, this study had a relatively large sample of men from four different countries, broadly representative of the general overweight male population in each country, and objective measurement of PA and sedentary time that enabled us to distinguish sitting from standing and other forms of PA. Our study also has some limitations. First, this study is a secondary analysis of data from a randomised controlled trial, which aimed to increase PA, reduce sedentary time, and improve diet. However, for the present analyses, the two groups (intervention and comparison groups) were merged, and a cohort analysis was performed. Thus, the extent to which causality can be inferred is less than that for a randomised controlled trial. Secondly, we cannot rule out the possibility that unmeasured confounders, such as changes in prescribed medications or sleep duration and quality, may have contributed to our observations. Dietary measures were self-reported and this may have attenuated the apparent association between changes in diet and changes in weight and cardiometabolic risk due to regression dilution bias effects. In addition, the dietary measures (whilst based on a validated tool) were modified to capture estimates of intake of sugary foods and these questions were not validated. No data is available on the reliability of the tool which was developed some years ago. The measures (presented as scores based on frequency of consumption) were indicative of intakes and cannot easily be translated to markers of nutrient status. The dietary data provides a broad view of intakes at a cross-sectional level and cannot be easily compared to other large survey data using different methodologies. The same dietary questions were used at both time points enabling perspectives on changes to be assessed, however further work on the development of dietary tools for the purpose is merited. Finally, it is important to recognise that the 7-day ‘snapshot’ measurements of PA made in this study may not have been fully representative of usual PA over the intervention period: any such error in assessment of the change in PA would have acted, via regression dilution bias, to underestimate the direct effect of PA change on change in cardiometabolic risk score.

## Conclusion

Changes in number of steps and diet, but not in standing time and sedentary time were associated with change in body weight. Furthermore, change in body weight and baseline cardiometabolic risk, explained most of the variance in the change in cardiometabolic risk, with changes in number of steps, but not sedentary time or standing time, and sugar intake making small contributions. Thus, the benefits of changing PA and diet on cardiometabolic risk seem to act largely via effects on changes in body weight in this study. The results suggest that lifestyle interventions aimed at reducing cardiometabolic risk should primarily focus on weight loss rather than PA, with PA and dietary improvement promoted for weight reduction and thus improved cardiometabolic health in overweight and obese middle-aged.

## Supplementary Information

**Additional file 1.**

## Data Availability

Data from the study are available for secondary analysis. Applications to access the data can be made by contacting Professor John Cleland, Director of the Robertson Centre for Biostatistics and Glasgow Clinical Trials Unit (John.Cleland@glasgow.ac.uk). Applicants are required to submit a brief proposal outlining their intended use of the data, but no genuine application from an appropriately qualified researcher will be refused. Access to the data will be given via a secure analytical platform.

## Abbreviations

PA: Physical activity
SED: Sedentary
BW: Body weight
CVD: Cardiovascular disease
EuroFIT: European fans in training
RCT: Randomised controlled trial
DINE: Dietary Instrument for Nutrition Education
PAR-Q+: Physical Activity Readiness Questionnaire-Plus questionnaire
BMI: Body mass index
HDL-C: High-density lipoprotein cholesterol
HbA1c: Hemoglobin A1c
